# Cavernoma of the cauda equina mimicking schwannoma: A case report

**DOI:** 10.1016/j.radcr.2025.01.043

**Published:** 2025-01-31

**Authors:** Reza Naseri, Maryam Haghighi-Morad, Zahra Mohammadi Manesh, Farahnaz Bidari Zerehpoosh, Hadi Vahedi, Hamidreza Ashayeri

**Affiliations:** a Department of Radiology, Loghman Hakim Hospital, Shahid Beheshti University of Medical Sciences, Tehran, Iran; b Student Research Committee, Shahid Beheshti University of Medical Sciences, Tehran, Iran; c Department of Pathology, Loghman Hakim Hospital, Shahid Beheshti University of Medical Sciences, Tehran, Iran; d Student Research Committee, Tabriz University of Medical Sciences, Tabriz, Iran; e Neuroscience Research Center (NSRC), Tabriz University of Medical Sciences, Tabriz, Iran

**Keywords:** Cavernous malformations, Cavernous hemangioma, Spinal cavernoma, Thoracolumbar spine, Intradural extramedullary, Magnetic resonance imaging

## Abstract

Cavernous malformations (CMs), also known as cavernomas or cavernous hemangiomas, are vascular lesions characterized by clusters of abnormally dilated blood vessels resembling a mulberry. In this report, we present the case of a 40-year-old man who presented with a one-year history of back pain radiating into both legs, with a preference for the left leg. An MRI initially suggested a schwannoma at the T12-L1 vertebral level, with a differential diagnosis that included meningioma—2 common intradural-extramedullary spinal tumors— with distinct management and prognostic implications. However, despite imaging findings consistent with a schwannoma, the final pathology revealed an intradural extramedullary (IDEM) cavernous hemangioma in the thoracolumbar region. Although cavernous hemangiomas are very rare in the spinal region, our case underscores the importance of considering them in the differential diagnosis of IDEMs in the thoracolumbar area.

## Introduction

Cavernous malformations, or cavernomas, are low-flow vascular lesions composed of abnormally dilated blood vessels with no muscle or adventitia tissue [1]. Cavernomas predominantly occur intracranially, and only about 5% are found in the spine, with the majority being extradural or intramedullary. Intradural-extramedullary (IDEM) cavernomas are particularly rare [2]. Spinal cavernoma can mimic the radiologic features of tumors such as schwannomas.

Schwannomas are a common type of intradural extramedullary tumor that can occur in the thoracic region. Intradural extramedullary schwannomas result from the local proliferation of Schwann cells in nerve sheaths [3]. This report presents a case of an IDEM cavernous hemangioma at the T12-L1 level that mimicked schwannoma. The lesion caused severe spinal cord compression, as evidenced by MRI, and was treated with surgery.

## Case presentation

A 40-year-old male patient presented with a one-year history of radicular low back pain radiating to both lower extremities with a preference for the left leg. The patient had undergone medical treatment and physical therapy to control the pain, with no improvement, and was subsequently referred to this center. The physical examination revealed no abnormalities, and the patient did not report any complaints of sphincter, sensory or motor dysfunction, or gait and balance issues. The patient has a past medical history of seizures, with the last occurrence being 4 years ago, and has been on sodium valproate and Carbamazepine (both taken twice daily) since childhood. He also has a past surgical history of knee surgery ten years ago.

**Fig. 1:**
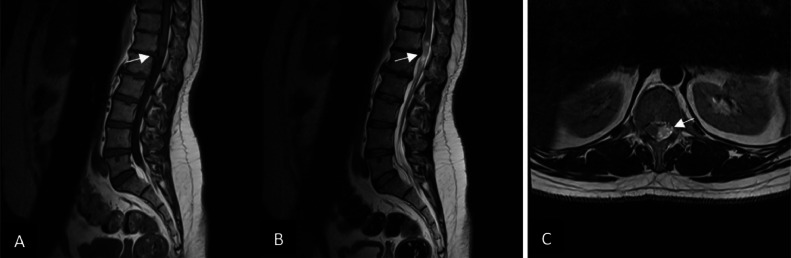
an oval intradural mass lesion is revealed on magnetic resonance imaging of the lumbar spine at the T12-L1 level, with a low signal on T1 W and a high signal on T2 W images marked by short arrows. There is obvious compression of conus medullaris by the mentioned mass.

**Fig. 2:**
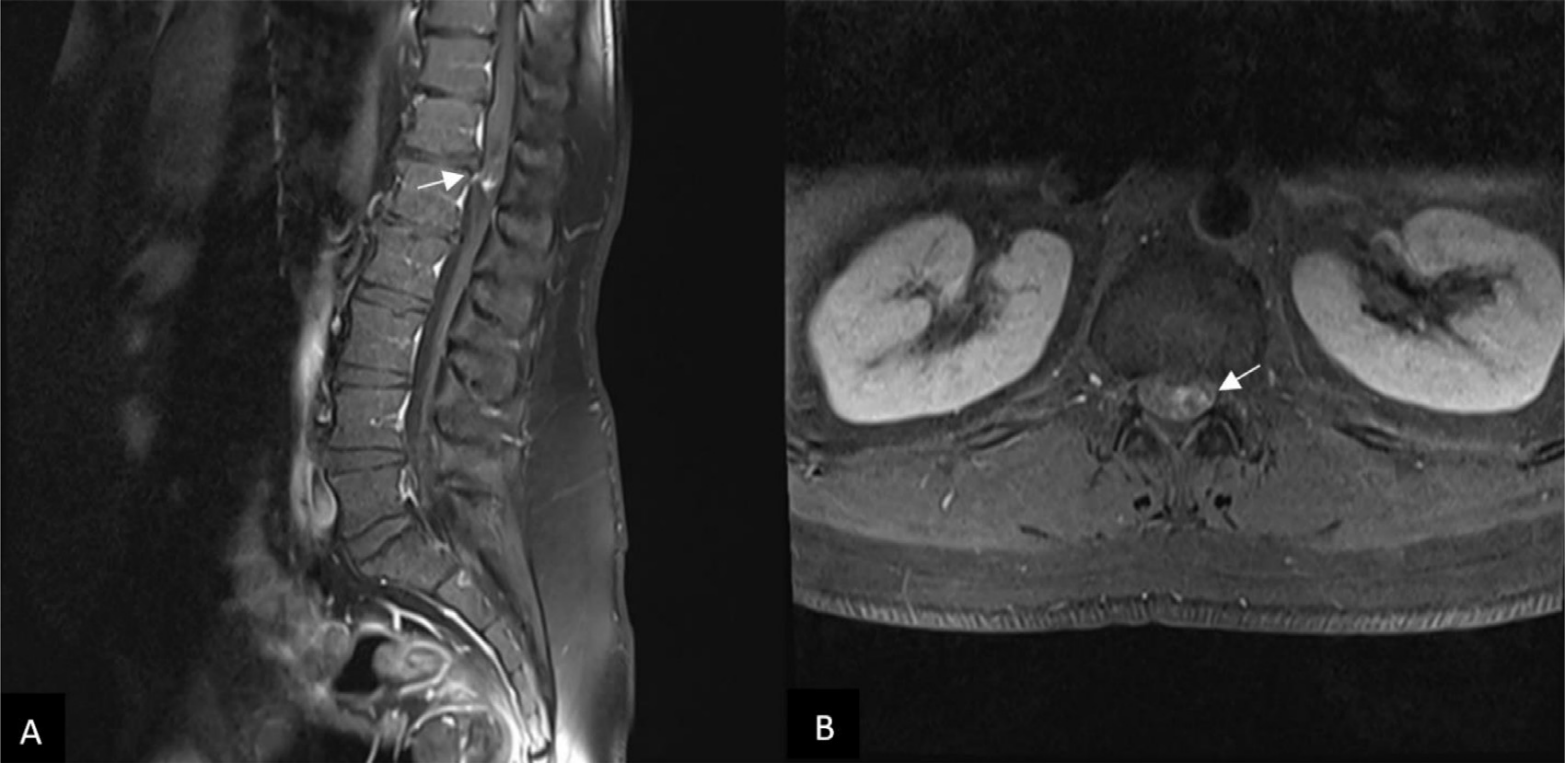
Postcontrast T1 W sagittal and axial images showed mild peripheral and nodular enhancement of the caudal part of the mentioned mass marked by short arrows.

Radiography of the thoracic spine showed spondylosis and degenerative changes at the thoracolumbar spine, more prominent in T12-L2 levels. MRI of the lumbosacral spine showed an intradural extramedullary enhancing mass lesion measuring 16×11 mm at the T12-L1 level, causing severe cord compression (Fig. 1, Fig. 2).

**Fig. 3:**
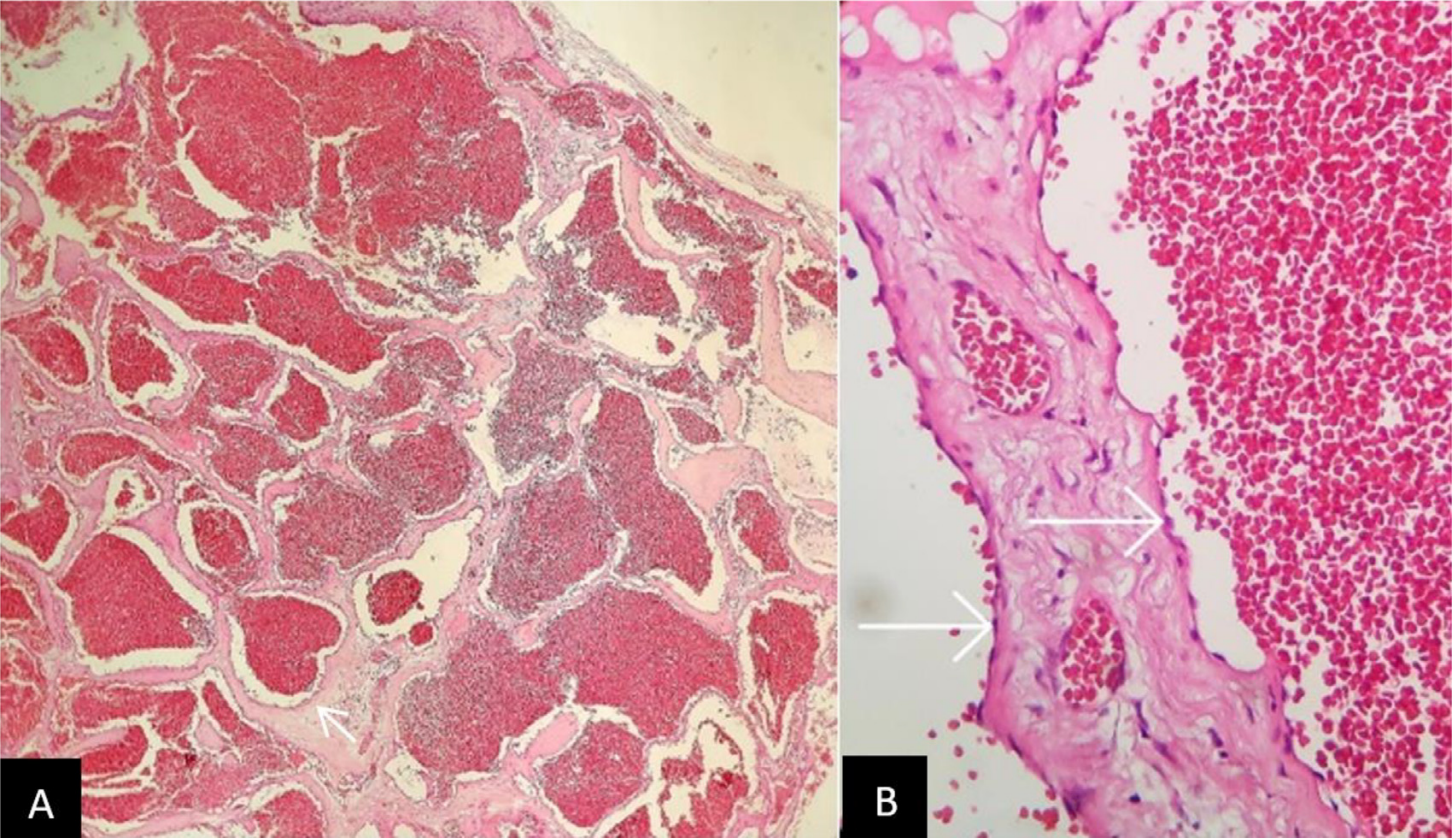
Dilated blood-filled vessels lined by flattened endothelium are shown in the microscopic examination of resected mass, marked by short arrows. (H&E stained, ×400).

According to higher prevalence, schwannoma and meningioma were considered in the differential diagnosis. Additionally, there is disc bulging with mild canal narrowing at T12-L1 and L1-L2 levels. Due to the patient's prolonged symptoms and failure of conservative management, he underwent total tumor resection and laminectomy at the T12-L1 level. Macroscopically a specimen with capsulated brown tissue measuring 1.5 *1 × 0.8 cm was received by the pathology department. Microscopic examination revealed a well-defined proliferation of dilated thin-walled vessels filled with blood and lined by flattened bland-looking endothelial cells, so the diagnosis of cavernous hemangioma was confirmed (Fig. 3). After surgery, the patient was discharged in good general condition. No evidence of radicular pain or neurological symptoms was identified during his follow-up.

## Discussion

This case of a cavernous hemangioma in the thoracolumbar region underscores the inherent challenges in differentiating spinal tumors, especially given the overlapping imaging characteristics with schwannomas and meningiomas [[2], [3], [4], [5]]. Such diagnostic dilemmas are critical, as accurate identification significantly influences the therapeutic approach and prognosis [4,5].

Spinal cavernoma is responsible for 5%-12% of spinal vascular malformations, and only 3% of them occur in intradural locations [6]. The lumbar spine is the most common location for spinal cavernoma. However, other places have also been reported [7]. Previously, 71 cases were reported in the literature, with 4 instances located in the thoracolumbar spine [8]. Among these, 50 cases involved nerve roots, while 21 were associated with other structures, such as the dura, or had unspecified origins [8]. IDEM spinal cavernoma is reported in a 20-79 age range group with a mean age of 49.7 [9]. Spinal cavernoma is also reported in younger individuals despite the rarity of the condition [4].

Spinal cavernoma can manifest different symptoms due to the compression of the spinal cord or hemorrhage. These manifestations can have either an acute or chronic course. Sensory impairments are the most reported clinical manifestation, followed by motor dysfunction and back pain [10]. Spinal cavernoma is usually seen in T1- and T2-weighted MRI images as well-defined, round, heterogeneous lesions resembling mulberry or popcorn. A hypointense rim can also surround the lesion in MRI due to the hemosiderin deposits resulting from previous bleeding [11]. Schwannoma is a benign soft tissue tumor that can represent a heterogenous appearance in both T1- and T2-weighted images mimicking the cavernoma [12]. MRI is a powerful tool in diagnosing cavernoma; however, sometimes cavernoma mimics other lesions. Thus, pathology remains the gold standard for diagnosing cavernoma. In the pathology, cavernoma represents a dilated vascular space with one endothelial layer [13].

Preoperative MRI is used for localization and defining the extension of the malformation [5]. Also, MRI is useful in investigating the presence of developmental venous anomalies, a finding that may affect the treatment plan for cavernoma [14]. The current recommended treatment for spinal cord cavernomas is resection of the lesion with surgery. This resection can result in the disappearance of the symptoms. Surgery is usually done 32 days after the occurrence of clinical signs and symptoms; however, there is a debate about the optimal timing of the surgery [15].

## Conclusion

Cavernomas, schwannomas, and meningiomas can occur within the spinal canal in similar locations (intra-, extradural, or mixed). Their MRI signals are iso- to hypointense on T1 and iso-to-hyperintense on T2-weighted images with well-defined margins. As mentioned above, these tumors overlap clinical findings and imaging characteristics with cavernomas, making their diagnosis complicated and challenging. Meningioma and schwannoma are 2 of the most common causes of IDEM spine lesions, while this is a relatively rare location for cavernoma compared to them. This case reports an overlap in the schwannoma and cavernoma diagnosis, which pathology confirmed. So, emphasis on characteristic imaging findings of each lesion and confirmatory histological examination remain the definitive methods of diagnosis.

## Patient consent

We claim and confirm that written, informed consent and full permission for publication, reproduction, broadcast and other use of our case scientific information (like past medical histories, present illness, photographs, recordings, other audio-visual material) was obtained from the patient and his first-degree family.

## CRediT authorship contribution statement

Conceptualization and project administration: Maryam Haghighi-Morad.

Investigation: Reza Naseri, Maryam Haghighi-Morad, Zahra Mohammadi Manesh, Farahnaz Bidari Zerehpoosh.

Writing - Original Draft: Hadi Vahedi, Hamidreza Ashayeri.

Writing—Review and Editing: Reza Naseri, Maryam Haghighi-Morad, Zahra Mohammadi Manesh, Farahnaz Bidari Zerehpoosh.

Visualization: Maryam Haghighi-Morad, Hamidreza Ashayeri.
